# Are leaf anatomical traits strong predictors of litter decomposability? Evidence from upper Andean tropical species along a forest successional gradient

**DOI:** 10.1007/s00442-025-05739-8

**Published:** 2025-06-24

**Authors:** Dennis Castillo-Figueroa, Juan M. Posada

**Affiliations:** Biology Department, Faculty of Natural Sciences, Universidad Del Rosario, Bogotá, Colombia

**Keywords:** Andean forests, Functional traits, Leaf anatomy, Leaf economic spectrum, Litter decay

## Abstract

Litter decomposability has been linked to "soft" traits of green leaves, but relationships with "hard" traits associated with leaf anatomy remain unexplored. Examining anatomical traits within the leaf economic spectrum may enhance our understanding of litter decomposability. In this study, we analyzed the relationships between leaf anatomical traits and decomposability at both species and community levels along a successional gradient of upper Andean tropical forests in Colombia. We conducted a reciprocal translocation field experiment with 15 upper Andean species in 14 permanent plots around Bogotá, collecting 2520 litterbags at four times (3, 6, 12, 18 months). Using a multiple regression model based on foliar traits, we estimated decomposability for the remaining 48 species that compose the plant community (63 species in total) in the studied successional gradient. We measured several leaf anatomical traits in all 63 species and calculated community-weighted means and functional diversity indices with the most effective anatomical predictors of decomposability. We found that thicker cuticles, larger vascular bundles, higher spongy mesophyll proportion, and lower palisade mesophyll proportion are related to low decomposability. Plant communities with thicker protective structures slow down decay rates, while large palisade tissues with cylindrical cells increase litter breakdown. Decomposability did not change along succession due to the balance between high functional evenness in secondary forests and high functional richness in mature forests. Despite potential circularity and interdependence between functional diversity metrics, our study provides novel insights into the anatomical basis of decomposability and community dynamics in successional gradients of upper Andean tropical forests.

**Supplementary Information:**

The online version contains supplementary material available at 10.1007/s00442-025-05739-8.

## Introduction

Leaves are the main organs for photosynthesis and play a crucial role in the functioning of terrestrial ecosystems (Wright et al. 2004; He et al. 2018; Ni et al. 2022). They contribute directly not only to key physiological functions such as carbon fixation, but also to respiration, transpiration, resource use, and plant growth (e.g., Kröber et al. 2015; Kuster et al. 2016; Rey-Sanchez and Posada 2019). Leaves have multiple functional traits, whose relationships have been extensively examined within the conceptual framework provided by the Leaf Economic Spectrum (LES) (Wright et al. 2004; Reich 2014; de la Riva et al. 2017). This framework accounts for approximately 75% of interspecific variation in traits related to carbon gain, water regulation, and nutrient use (Wright et al. 2004; Díaz et al. 2016; Esquivel et al. 2020), and explores selective pressures that give rise to trade-offs between the acquisition and conservation of resources (Reich 2014). Consequently, the LES has been extended to encompass significant ecosystem processes, such as water use efficiency and productivity (He et al. 2018), as well as nutrient cycling and litter decomposability (Bakker et al. 2011; de la Riva et al. 2019; Esquivel et al. 2020).

Previous investigations of litter decomposability have focused on “soft” leaf functional traits, which are more readily measurable, including Leaf Mass per Area (LMA), Leaf Nitrogen Content (LNC), and Leaf Dry Matter Content (LDMC) (Santiago 2007; Bakker et al. 2011; Pérez-Harguindeguy et al. 2015; Garnier et al. 2016; Li et al. 2023). However, the LES is expected to be the result of fundamental trade-offs in form and function that are closely related to the anatomy of leaves. Specifically, it has been suggested that the LES is governed by trade-offs between allocation to structural tissues and to metabolic (‘liquid phase’) processes (Shipley et al. 2006; Onoda et al. 2017). Thus, measuring the anatomical traits that underpin the LES could improve our understanding of litter decomposability.

Trade-offs between traits are partially expressed in the relationships between epidermis, cuticle, vascular bundles, and mesophyll tissues (Onoda et al. 2012; Somavilla et al. 2014; Harrison et al. 2021). Within leaf anatomical structures, thicker cuticles and epidermis serve as the initial physical barrier for plants, representing crucial defenses that enhance leaf protection and longevity (Onoda et al. 2012; Harrison et al. 2021). The epidermis also plays a key role in light transmission, and it holds the stomata that control CO_2_ diffusion (Verboven et al. 2015; He et al. 2018), transpiration, and heat balance (Liu et al. 2021). Thus, given their function as a barrier, the epidermis and cuticle should also influence decay rates by reducing the entry of decomposers (Zukswert and Prescott 2017; Onoda et al. 2012). In addition, larger spongy mesophyll thickness has been related to lower water use efficiency because of higher water loss by evaporation (He et al. 2018). Nevertheless, the spongy mesophyll is positively related to gross primary production at the community level, as it enhances gas transportation and maximizes carbon assimilation (Somavilla et al. 2014; He et al. 2018). Thicker palisade mesophyll is associated with higher photosynthetic capacity and greater pigment content, which improves light capture and overall plant growth (Terashima et al. 2011; Chen et al. 2015; Kröber et al. 2015; Harrison et al. 2021). Therefore, the ratio of palisade tissue thickness to spongy tissue thickness serves as a good proxy for leaf gas exchange and photosynthetic efficiency (Somavilla et al. 2014; He et al. 2018; Harrison et al. 2021; Liu et al. 2021). Lastly, vascular bundles are conductive tissues responsible for the transport of critical resources such as water, nutrients, sugars, and amino acids, but also, they play a fundamental role in the provision of mechanical support to the leaves (Lucas et al. 2013; Ni et al. 2022).

Although the LES has been mainly investigated from the perspective of the economics of carbon gain (Wright et al. 2004; Harrison et al. 2021; Onoda et al. 2017), the same leaf traits can also predict decomposability due to ‘after life effects’ (Santiago 2007; Bakker et al. 2011; de la Riva et al. 2019; Esquivel et al. 2020). This relationship should be reflected in the connection between anatomy and litter decomposability. For instance, some evidence suggests that mesophyll cells decompose faster than upper and lower epidermis tissues due to their chemical composition (Pavlović et al. 2020). Specifically, mesophyll contains higher nitrogen in the form of proteins, while epidermis and cuticles consist of waxes, fats, and cutins that shield leaves from degradation (Pavlović et al. 2020). Other anatomical structures in leaves, such as vascular bundles, hypodermis, and air spaces, may also play a significant role in decomposability but they require further investigation (Harrison et al. 2021).

Leaf traits can change along environmental gradients, influenced by state factors such as light, soil nutrients, temperature, and water availability (Siefert et al. 2015; An et al. 2021; Xu et al. 2021; Galviz and Valerio 2021; Ni et al. 2022). These environmental factors can vary widely with forest succession, resulting in changes in leaf traits within the community (Poorter et al. 2021a). This is particularly relevant given that secondary forests are becoming one of the most common cover in the tropics due to the pervasive landscape transformation (Chazdon et al. 2014). Nonetheless, research on changes in leaf anatomical traits at the plant community level across successional gradients in tropical regions is scarce, and their potential implications for litter decomposability remain largely unexplored.

One of the tropical regions undergoing significant transformation is located in the Andean montane forests surrounding the Colombian capital city, Bogotá, situated in Northern South America. Despite this place is the highest tropical Andean forest belt and represents a region with an extraordinary beta-diversity (Calbi et al. 2021; Hurtado-M et al. 2021; Cedillo et al. 2023), and endemism (Myers et al. 2000; Myster 2021; Castillo-Figueroa et al. 2024), less than the 20% of the original cover remains because of the expansion of agricultural activities since the Spanish colonization and subsequent urbanization (Etter and Wyngaarden 2000; Etter et al. 2021). Recent studies, however, have shown an increase in forest regeneration resulting from land use changes over the last few decades (Rubiano et al. 2017; Calbi et al. 2020), leading to a mosaic of forests with diverse successional pathways. Although some efforts have been made to study the functional recovery of upper Andean tropical forests (Castillo-Figueroa et al. 2023, 2025; Castillo-Figueroa and Posada 2025), our understanding about how leaf functional traits relate to different components of the carbon cycle along successional gradients remains limited (Castillo-Figueroa 2021). Analyzing leaf anatomical traits along these Andean successional forests can be useful to better understand how plant communities respond to forest regeneration and whether these traits are good predictors of litter decomposability.

Successional theory suggests that leaf functional traits become more conservative as succession progresses (e.g., high LDMC, LMA, and C:N ratio; Pinho et al. 2018; Poorter et al. 2021b). This pattern arises because the successional gradient reflects a shift from environments with high nutrient and light availability to conditions of lower resources, with reduced light and nutrients (Chua and Potts 2018; Poorter et al. 2023). Such changes drive species turnover, potentially shifting the community's strategy from acquisitive (high decomposability) to conservative (low decomposability) (Poorter et al. 2021b). Therefore, the decline in community-level decomposability along the gradient should correspond to the dynamics of community structure influencing decay rates. Based on this, one would expect a decrease in the functional diversity of leaf anatomical traits that most strongly influence decomposability as succession advances, leading to lower decay rates in old-growth forests.

In this study, we investigate the relationships between leaf anatomy and litter decomposability along a successional gradient in tropical Andean montane forests of Colombia. Based on functional traits from different leaf tissue types, our objectives were to: (1) analyze the relationships between leaf anatomical traits and litter decomposability at species level, (2) assess how leaf anatomical traits influence community litter decomposability and, (3) evaluate changes in community litter decomposability and the functional diversity of anatomical traits that best predict decay rates along the successional gradient in tropical Andean montane forests. We hypothesized that the size (thickness and area) and fractions (percentages) of tissue types such as mesophylls, epidermis, vascular bundles, and cuticles will exhibit strong associations with decomposability due to their links to physiological processes related to resource acquisition and leaf defenses that “carry over” once leaves die (H1). We also predicted that forests with higher community weighted thickness or area in protective and conductive tissues, such as cuticles, epidermis, and vascular bundles, but with lower thickness of photosynthetic tissues, including spongy and palisade mesophylls, will display lower decay rates (H2). Finally, we predicted that community litter decomposability will decrease with advancing succession because plant communities will converge towards more conservative strategies. This will result in mature forests exhibiting lower functional richness, evenness, divergence, and dispersion in anatomical traits related to decomposability (H3).

## Materials and methods

### Study area

The Andean region encompasses 24.5% of Colombia's territory (Etter and Wyngaarden 2000). This region stands as the economic epicenter of the country and is home to approximately 70% of its population (DANE 2018). This study was conducted within the Eastern Colombian Andes, specifically in the Cundiboyacense high plains, a highly transformed region situated within the tropical Andean montane forests (Etter et al. 2021). In this region, dominant human activities include agriculture, cattle ranching, urban development, and mining (Montañez et al. 1994; Etter and Wyngaarden 2000; Mendoza and Etter 2002). The area surrounding the capital city of Bogotá experiences an average annual atmospheric temperature of 14 °C, with mean annual precipitation ranging from 600 mm in central valleys to 1200 mm in the western regions (Clerici et al. 2016). The high plains display a bimodal precipitation pattern, characterized by two rainy periods from April to June and from October to December, separated by two drier seasons from January to March and July to September (IDEAM 2024).

Our research took place on 14 permanent plots (20 × 20 m) established as part of the "Rastrojos" project (a larger network consisting of 36 20 × 20 m plots and eight 50 × 50 m plots, see acknowledgments). These 14 plots were originally set up in 2013 within private properties/reserves in upper Andean tropical forests at four distinct sites, at elevations ranging from 2685 to 3140 m (Hurtado-M et al. 2021; Castillo-Figueroa et al. 2023; Castillo-Figueroa and Posada 2025). The four study sites were: Torca (4° 48′ 48.674" N, 74° 0′ 58.527" W, 2708-2965 m), Tabio (4° 55′ 33.961" N, 74° 6′ 47.225" W, 2685-2821 m), Guasca (4° 47′ 20.318" N, 73° 54′ 31.812" W, 3085-3140 m), and Guatavita (4° 56′ 9.716" N, 73° 53′ 54.237" W, 3028–3035 m) (Fig. S1).

At these sites, the 14 permanent plots were evenly distributed between secondary (seven plots) and mature forests (seven plots) across the study locations of Tabio (two per successional stage), Guasca (two per successional stage), Torca (three in mature forest and one in secondary forest), and Guatavita (two in secondary forest). Mature and secondary forests were categorized based on structural attributes such as tree height, tree density, basal area, and species composition, according to previous studies (Hurtado-M et al. 2021; Castillo-Figueroa et al. 2023). Among the plant families found in these plots, Ericaceae, Melastomataceae, Cunoniaceae, Primulaceae, and Asteraceae collectively constitute 56% of all individuals with a basal diameter exceeding 5 cm. The most dominant genera include *Miconia*, *Weinmannia*, *Cavendishia*, *Myrsine*, and *Myrcianthes*, which together account for 51% of all individuals. In the study area, we identified a total of 63 species of shrubs and trees, with the dominant species being *Weinmannia tomentosa* Linnaeus filius 1782, *Cavendishia bracteata* Hoerold 1909, *Miconia ligustrina* Triana 1872, *Miconia squamulosa* Triana 1872, and *Myrcianthes leucoxyla* McVaugh 1963 (Clerici et al. 2016; Castillo-Figueroa et al. 2023).

### Litter decomposition set-up

We set-up a reciprocal translocation field decomposition experiment with 15 upper Andean tropical species in the 14 permanent plots between October 2021 and April 2023 (Castillo-Figueroa et al. 2025). These species belonged to 15 families and 12 orders and were selected based on two criteria: (1) the dominance of the species (Table S1), considering that the most abundant ones would contribute more significantly to litter on the forest floor (Salinas et al. 2011; Esquivel et al. 2020); and (2) the representation of species in the functional trait space within the plots (Canessa et al. 2021), based on previous analyses (Table S2; Castillo-Figueroa et al. 2025).

We prepared three independent litterbeds per plot for a total of 42 litterbeds. Each litterbed was placed directly on the forest floor, trying to minimize disturbance to the soil and with a minimal distance of 5 m between them. We avoided forest gaps, topographic depressions, and very irregular soil conditions. Each litterbed was made of 60 litterbags with four litterbags per species arranged clockwise according to consecutive harvesting times (3, 6, 12, and 18 months). The distance between litterbags of different species (15 taxa) was 10 cm. Litterbags were made of fiber glass (10 × 15 cm) with a mesh size of 2 mm that contained ca. 2 g of oven dried litter (60 ºC). This resulted in a total of 2520 litterbags in the decomposition experiment (15 species × 4 times × 42 litterbags).

We collected one litterbag per species from each litterbed at each of the four harvesting times. In the laboratory, the contents of the litterbags were sorted to separate litter from fine roots, forbs, mushrooms, mineral soil particles, and soil fauna. The litter material was gently cleaned with a brush to remove mineral soil particles and then was oven-dried at 60 °C for 72 h and weighed using a precision scale with a sensitivity of 0.1 mg (LX 220A scs) to determine both initial and final weights.

### Foliar functional traits

We measured green leaf traits for all 63 species present in the 14 permanent plots, collecting three leaves from three individuals per species (Posada et al. unpublished). Leaves were collected from the sunny canopy using a branch cutter, within the permanent plots, following standardized protocols (Pérez-Harguindeguy et al. 2013). We measured one-sided leaf area by scanning the leaves and analyzing the resulting images using ImageJ (Schneider et al. 2012, https://imagej.nih.gov/ij/). To determine maximum fresh mass (g), we rehydrated leaves in plastic bags filled with moist paper for 24–48 h at a low temperature (4 °C) in the dark, following the complete rehydration method proposed by Garnier et al. (2001). Each leaf sample was then dried in the oven at 60 °C for 72 h to determine its dry mass (g). From these measurements we obtained Leaf Mass per Area (LMA, g m^−2^), its reciprocal Specific Leaf Area (SLA, cm^2^ g^−1^), and Leaf Dry Matter Content (LDMC, mg g^−1^). We also measured Leaf Nitrogen Content (LNC, mg g^−1^), Leaf Carbon Content (LCC, mg g^−1^) and Carbon-to-Nitrogen content ratio (C:N) using an Elemental Analyzer (FlashSmart™ Thermo Fisher Scientific, USA). Finally, we measured leaf photosynthetic capacity (A_max_, nmol g^−1^ s^−1^) by exposing leaves to a saturating photosynthetic photon flux density (PPFD, μmol m^−2^ s^−1^) using an infrared gas analyzer (LiCor, LI-6400XT) equipped with a red-blue light source (LiCor, 6400-02B).

### Leaf anatomical traits

For leaf anatomical traits, we measured the cuticle, epidermis, hypodermis, palisade mesophylls, spongy mesophylls, vascular bundles, and air space from the 63 species present in the 14 permanent plots (Fig. S2, Table 1; Castillo-Figueroa 2025). To do this, we collected three sun leaves from three individuals for each species directly in the permanent plots. Then, we cut rectangular sections (1.0 × 0.5 cm) of the leaf lamina, including the midrib, which were then fixed in Formalin-Acetic acid-Alcohol (FAA, 50 ml 38% formalin, 50 ml glacial acetic acid, 70% ethanol 900 ml) (He et al. 2018). Leaf tissues were gradually dehydrated in an ethanol series (50%, 70%, 85%, 95%, 100%) and were infiltrated with hot paraffin (He et al. 2018; Harrison et al. 2021).

**Table 1:** Description of leaf anatomical traits based on thickness measurements, percentage of tissues (relative to leaf thickness), tissue ratios, and cell shapes

Leaf anatomical trait	Acronym	Unit
Thickness measurements		
Adaxial cuticle thickness	AdCT	µm
Abaxial cuticle thickness	AbCT	µm
Adaxial epidermis thickness	AdET	µm
Abaxial epidermis thickness	AbET	µm
Adaxial hypodermis thickness	AdHT	µm
Palisade mesophyll thickness	PMT	µm
Spongy mesophyll thickness	SMT	µm
Vascular bundle diameter	VBD	µm
Leaf thickness	LT	µm
Air space	AS	µm^2^
Vascular bundle area	VBA	µm^2^
Leaf total area	LTA	µm^2^
Leaf area cell tissue	LACT	µm^2^
Percentage of tissues (relative to LT)		
Percentage of Adaxial cuticle thickness	%AdCT	%
Percentage of Abaxial cuticle thickness	%AbCT	%
Percentage of Adaxial epidermis thickness	%AdET	%
Percentage of Abaxial epidermis thickness	%AbET	%
Percentage of Adaxial hypodermis thickness	%AdHT	%
Percentage of Palisade mesophyll thickness	%PMT	%
Percentage of Spongy mesophyll thickness	%SMT	%
Percentage of Air space	%AS	%
Tissue ratios		
Adaxial epidermis thickness / Palisade mesophyll thickness	AdET/PMT	Adimensional
Adaxial epidermis thickness/ Spongy mesophyll thickness	AdET/SMT	Adimensional
Palisade mesophyll thickness/ Spongy mesophyll thickness	PMT/SMT	Adimensional
Adaxial epidermis thickness/ Abaxial epidermis thickness	AdET/AbET	Adimensional
Adaxial cuticle thickness/ Abaxial cuticle thickness	AdCT/AbCT	Adimensional
Cell shapes		
Adaxial epidermis cell width	AdEw	µm
Adaxial epidermis cell long	AdEl	µm
Palisade mesophyll cell width	PMw	µm
Palisade mesophyll cell long	PMl	µm
Spongy mesophyll cell width	SMw	µm
Spongy mesophyll cell long	SMl	µm
Abaxial epidermis cell width	AbEw	µm
Abaxial epidermis cell long	AbEl	µm
Aspect ratio of Adaxial epidermis cells	As AdEc	Adimensional
Aspect ratio of Palisade mesophyll cells	As PMc	Adimensional
Aspect ratio of Spongy mesophyll cells	As SMc	Adimensional
Aspect ratio of Abaxial epidermis cells	As AbEc	Adimensional

Transverse sections of the leaf were cut with a Leica RM2255 microtome to obtain a flat surface for the subsequent estimation of cell fractions. The anatomical cuts of the leaves had a thickness between 5 and 7 µm. Lignified tissues were red-stained using Safranin and non-lignified tissues were stained with Toluidine blue, and then mounted in slides (Bancroft and Cook 1984; He et al. 2018). Then, we took photographs using a reflected (episcopic) light illumination microscope (Leica DM 750) equipped with a digital camera (Leica MC170 HD) with a 10X objective lenses. Calibration was done with a Nikon micrometer (MBM 11100 stage micrometer type A) with a 1 mm ruler with 0.01 mm graduati​o​n​s (i.e., 10 μm). For each individual, we took 10 focused images of different leaf segments for a total of 30 images per species.

Measurements of anatomical traits were conducted in the software ImageJ (Schneider et al. 2012, https://imagej.nih.gov/ij/). From each anatomical image, the thickness of the cuticles, epidermis, hypodermis (if any), palisade and spongy mesophylls, and vascular bundles were measured (Fig. S2, Table 1), resulting in a total of 10 measurements per leaf and 30 per species for each trait. In the case of epidermis and cuticles, both adaxial and abaxial thickness were measured. Based on these thickness measurements, we also calculated percentages (%) of each anatomical trait relative to the total leaf thickness. To measure % air spaces, we selected 10 rectangles that were 100 µm wide by the total leaf thickness and calculated the area with and without leaf tissue (Fig. S2, Table 1; Harrison et al. 2021). We enhanced these sections for color and contrast, and then transformed them into black (cell tissues) and white (air space) in the software ImageJ (Harrison et al. 2021). Ratios of adaxial epidermis/palisade mesophyll thickness, adaxial epidermis/spongy mesophyll thickness, palisade/spongy mesophyll thickness, adaxial/abaxial epidermis thickness, and adaxial/abaxial cuticle thickness were obtained per image (Table 1). We also measured the length and width of 10 random cells from both the adaxial and abaxial epidermis, palisade and spongy mesophylls to compute cell aspect ratios (i.e., width/length) (Table 1, Fig. S2). Higher values correspond to cells with more circular or squared shapes, while lower values correspond to more cylindrical or rectangular shapes. Given that this study focused on interspecific variations, we did not account for intraspecific variability. Therefore, all measurements of anatomical traits were averaged at the species level.

### Statistical analysis

To estimate decay rates coefficients per year (K rate y^−1^), we followed the negative exponential model (Jenny et al. 1949; Olson 1963), using the mass loss obtained for each species at the harvesting time of 12 months:

Y_t_ = Y_o_ * exp ^(-kx)^

where *Y*_*t*_ represents the remaining dry weight of leaf litter after time *t*, *Y*_*o*_ indicates the initial dry weight of leaf litter (maximum Y value), *k* depicts the decomposition constant that determines the steepness of the curve, and *x* is the time for decomposition (years) (Berg and McClaugherty 2020). With this equation, we obtained mean decomposition constants for each species (K rate y^−1^, species = 15) as follows:

$$\frac{\text{ln}(Yt/Y0)}{x}=-k$$

We obtained Spearman correlations (rho coefficients) between anatomical traits and decay rates (H1), as Shapiro–Wilk test for multivariate normality indicated a deviation from normality (P < 0.01). Correlations were divided in four separate plots: thickness measurements, percentage of tissues (relative to the leaf thickness), tissue ratios, and cell shapes (Table 1). To evaluate the association between leaf anatomical traits, foliar traits, and decomposability, we conducted a Principal Component Analysis (PCA) using functional traits and decay rates as variables, with species as data points (n = 15), following the approach outlined by Bakker et al. (2011). Given the limited number of species, we ran a PCA using the variables that were significantly correlated with decay rates in the Spearman correlation plots, excluding those with high intercorrelation (rho > 0.85). Additionally, we included the LMA, LDMC, and C:N ratio measured for these species.

To analyze the community-level response (H2), we did a multiple linear regression model between decay rates and the four foliar traits (i.e., SLA, LNC, LCC, A_max_) measured in the 15 species that were part of the decomposition experiment (r^2^ = 0.71, see Results). We selected these traits as they are the baseline of the LES (Wright et al. 2004; Reich 2014), which is strongly related to decomposition (Santiago 2007; Bakker et al. 2011; de la Riva et al. 2019). Then, we used the parameters of this multiple regression model to estimate decay rates for the remaining 48 species in our plots with their foliar traits measured (Table S3). Following, we calculated Community-Weighted Mean (CWM) of decomposability per plot (CWM K rate y^−1^) by weighting each estimated decay rate value by the biomass of each individual species in the plot (Castillo-Figueroa et al. 2023). This assumption was grounded on the premise that plot decay rates are primarily influenced by the species with the highest biomass, rather than the overall number of species in the community (i.e., mass ratio effect; Garnier et al. 2004; de la Riva et al. 2019). We then estimated biomass weighted CWM of each anatomical trait and performed reduced major axis regressions analyses with 95% bootstrapped confidence intervals of the slopes (n = 1999) between each one of these traits and CWM K rate y^−1^. Aboveground Biomass (AGB) in each plot was calculated by using allometric equations developed for tropical Andean montane forests (Sierra et al. 2007; Pérez and Díaz 2010).

Lastly, to analyze changes in community litter decomposability and the functional diversity of anatomical traits that best predict decay rates along the successional gradient (H3), we did a stepwise multiple linear regression model with the traits that exhibited significant relations with CWM K rate y^−1^. We used AGB as a proxy for succession, as it is a widely accepted indicator of forest recovery (Garnier et al. 2004, 2016; Lohbeck et al. 2015; Poorter et al. 2021a). To prevent multicollinearity between anatomical traits, we assessed the Variance Inflation Factor (VIF) for each trait. We included traits with VIF values below 1.1, to prevent collinearity between our variables (Neter et al. 1990). We assessed the best model based on the Adjusted r^2^ and Root Mean Squared Error (RMSE). Adjusted r^2^ offers a relative measure of model fit, considering the number of predictors and penalizing the inclusion of irrelevant variables to prevent overfitting (James et al. 2013), while RMSE provides a direct measure of prediction accuracy by quantifying the average deviation of predicted values from actual values in the same units as the dependent variable (Chatterjee and Hadi 2015). This enabled us to identify the anatomical traits that best predicted CWM K rate y^−1^ to compute functional richness (i.e., volume of the functional trait space occupied by the community), evenness (i.e., species abundances distribution across the functional trait space), divergence (i.e., deviance of abundance from the center of gravity of the functional space), and dispersion (i.e., abundance-weighted distance of species from the trait space centroid) (Mason et al. 2005; Villéger et al. 2008; Schmera et al. 2023). To standardize functional traits, we transformed the trait values into a common scale using Z-scores, with a mean of 0 and a standard deviation of 1 (Zar 1999). We regressed AGB on CWM K rate y^−1^ as well as on each of the functional diversity indices to analyze the dynamics of community structure underlying decomposability along the successional gradient. Bar plots showing mean values with 95% confidence intervals for each variable in mature and secondary forests were included to provide complementary information. All the analyses were conducted in PAST 4.14 (Hammer 2001) and JASP 0.14.1.0 (JASP TEAM 2023) and Fdiversity (Casanoves et al. 2011).

## Results

### Anatomical traits

Leaf anatomy from the 15 plant species of the experiment exhibited high variation in all the tissues measured. Average values and standard deviations of leaf tissues across these plant species are presented in Table S4. AdCT varied ca eight folds (2.68 to 22.52 µm); AdET ca five folds (8.70 to 44.11 µm); AdHT, when present, ca six fold (17.54 to 111.73 µm); PMT ca two folds (53.79 to 144.04 µm); SMT ca ten folds (56.90 to 567.48 µm); AbET ca three folds (7.38 to 22.58 µm); AbCT ca four folds (2.30 to 10.44 µm); AS ca 11-folds (702.88 to 8202.49 µm^2^); VBA ca 46-folds (1287 to 59,821.36 µm^2^); VBD ca seven folds (41.99 to 331.40 µm), and LT ca five folds (157.62 to 832.06 µm) (Table S4).

**Fig. 1:**
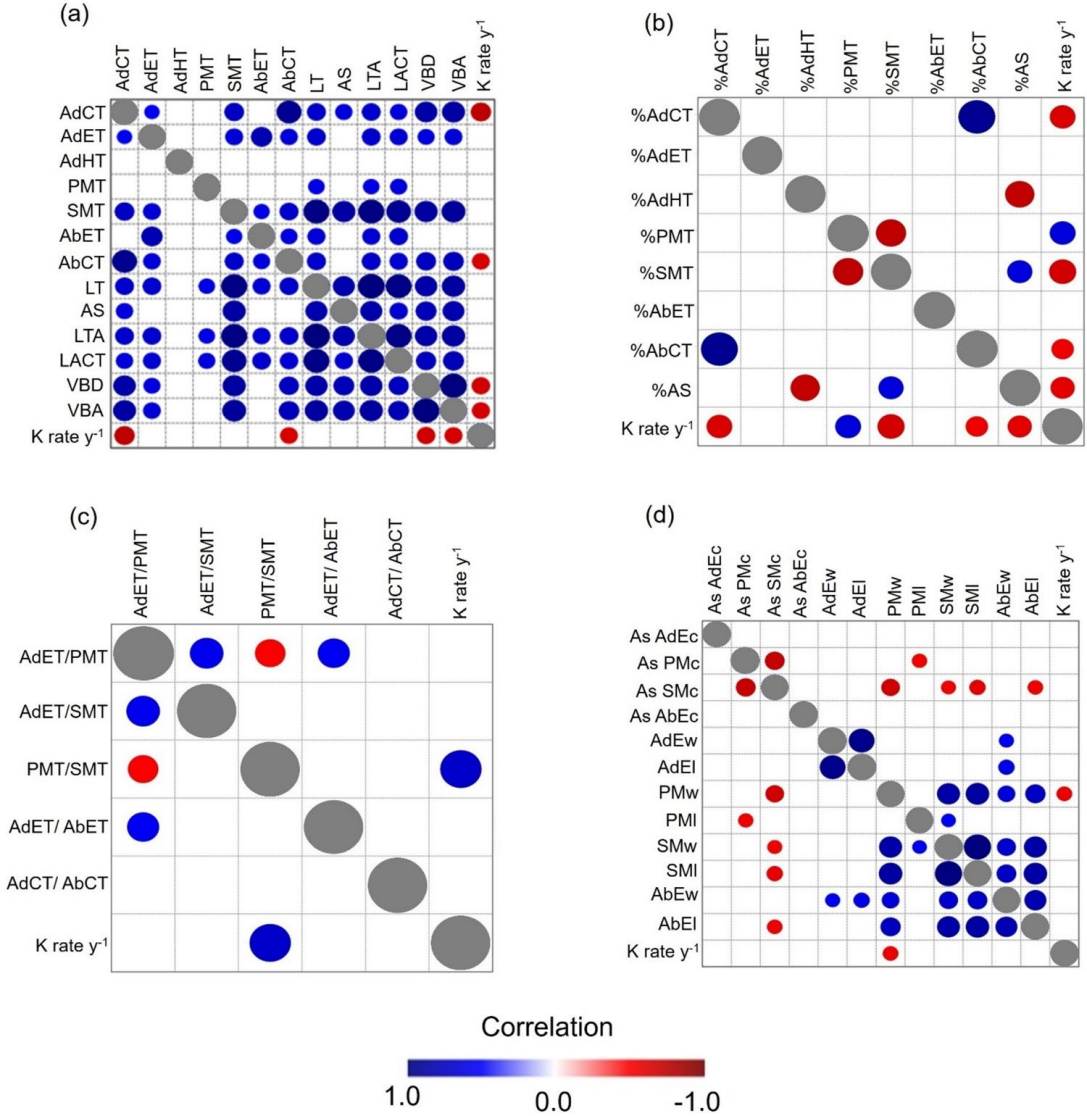
Spearman correlations between anatomical traits and **a** decomposition considering absolute thickness measurements **b** percentage of the tissues **c** ratios between tissues **d** and cell shapes. Positive correlations are indicated in blue, while negative correlations are represented in red. The legend color corresponds to the correlation coefficient and its gradient. The color intensity varies proportionally with the correlation coefficients from -1 to 1. In cases where the indicator is not statistically significant (P > 0.05), it is symbolized with blank. Acronyms can be found in Table 1

Anatomical leaf traits were positively correlated between them in 52 of the 78 possible correlations (66.7%), while they were not correlated in the remaining 26 cases (33.3%) (Fig. 1a). AdHT was the only trait that was not correlated to any mainly because it was only present in a few species. Lastly, PMT thickness was not correlated to the other traits, except for LT, LTA y LACT (Fig. 1a).

### Correlations between leaf anatomy and species decay rates

We found significant negative correlations between decay rate and the size of the AdCT (P = 0.002, rho = − 0.75, n = 15), AbCT (P = 0.011, rho = − 0.65, n = 15), VBA (P = 0.010, rho = − 0.65, n = 15), and VBD (P = 0.007, rho = − 0.68, n = 15) (Fig. 1a). We also found negative correlations between K rate y^−1^ and %AdCT (P = 0.013, rho = − 0.64, n = 15), %SMT (P = 0.008, rho = -0.67, n = 15), % AbCT (P = 0.036, rho = -0.55, n = 15), and %AS (P = 0.021, rho = − 0.60, n = 15), while K rate y^−1^ was positively related to %PMT (P = 0.012, rho = 0.64, n = 15) (Fig. 1b). Only the ratio PMT/SMT was related to K rate y^−1^ (P = 0.005, rho = 0.70, n = 15) and the relationship was positive (Fig. 1c). Regarding cell size and shape, we only found a negative relationship between PMw (P = 0.035, rho = − 0.55, n = 15) and K rate y^−1^ (Fig. 1d).

**Fig. 2:**
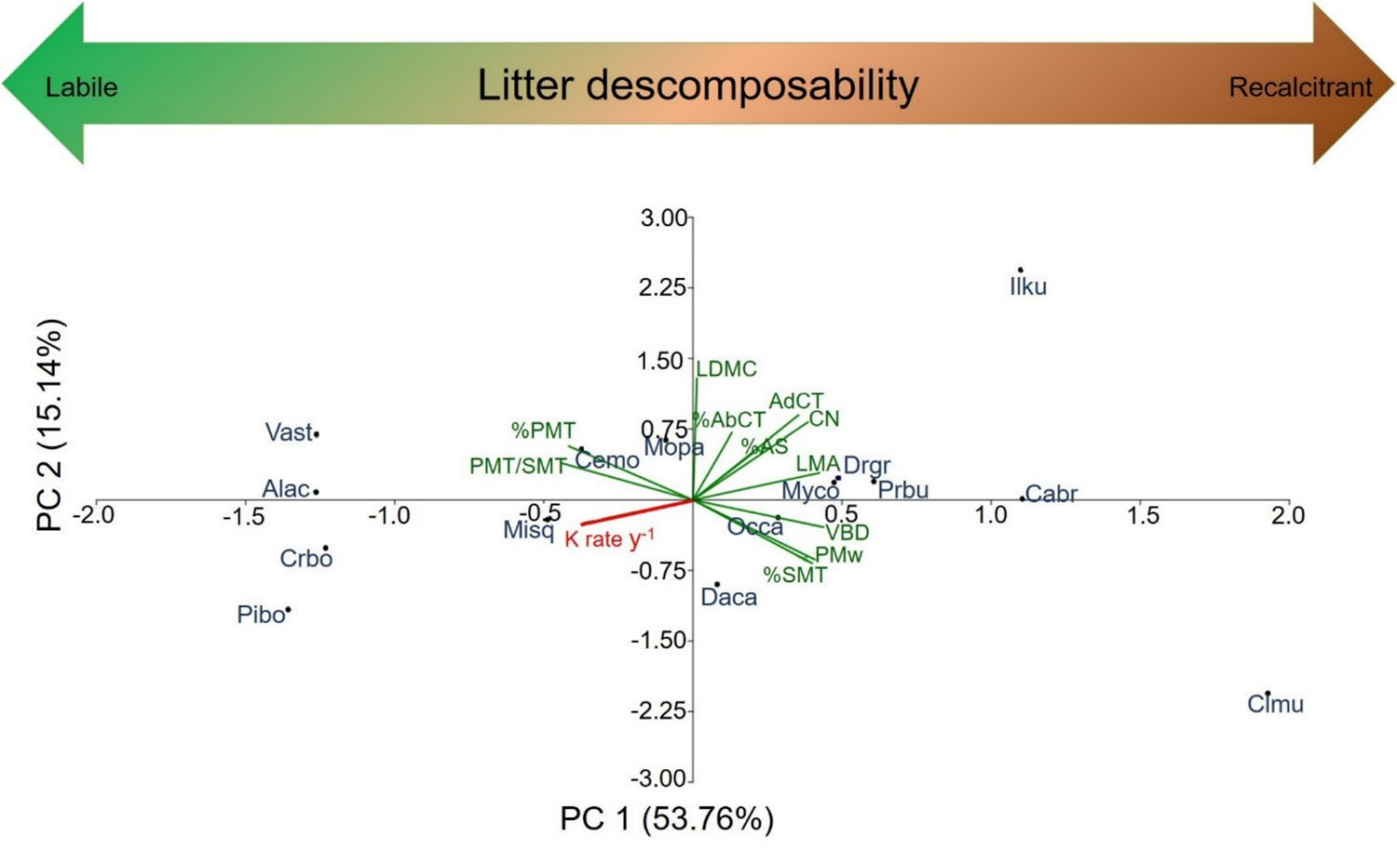
Principal Component Analysis between anatomical traits and decay rates (K rate y^−1^, in red). The leaf functional traits are shown in green: %Abaxial cuticle thickness (%AbCT), Adaxial cuticle thickness (AdCT, µm), Pallisade mesophyll cell width (PMw, µm), %Pallisade mesophyll thickness (%PMT), %Spongy mesophyll thickness (%SMT), %Air space (%AS), Vascular bundle diameter (VBD, µm), Pallisade/Spongy ratio (PMT/SMT), Leaf Mass per Area (LMA, g m^−2^), Leaf Carbon: Nitrogen ratio (C:N), and Leaf Dry Matter Content (LDMC, mg g^−1^). The 15 litter species are shown in blue: *Alnus acuminata* (Alac), *Cavendishia bracteata* (Cabr), *Cedrela montana* (Cemo), *Clusia multiflora* (Clmu), *Croton bogotanus* (Crbo), *Daphnopsis caracasana* (Daca), *Drimys granadensis* (Drgr), *Ilex kunthiana* (Ilku), *Miconia squamulosa* (Misq), *Morella parvifolia* (Mopa), *Myrsine coriacea* (Myco), *Ocotea calophylla* (Occa), *Piper bogotense* (Pibo), *Prunus buxifolia* (Prbu), *Vallea stipularis* (Vast)

Multidimensional relationships between anatomical and foliar traits, with decay rates were analyzed with the PCA (Fig. 2). The first axis explained 53.76% of the variation, and was positively related to LMA, C:N, VBD, AdCT, PMw, %SMT and %AS, but it was negatively related to %PMT, PMT/SMT and decay rates. The second axis explained 15.14% of the variation and was positively related to %AbCT and LDMC (Fig. 2). Species are clustered in this multivariate foliar and anatomical space according to their decomposability. Species with high decay rates, such as *Piper bogotense* (Pibo) and *Croton bogotanus* (Crbo), were positioned at the left extreme of the first axis, while species that exhibit low decay rates, such as *Cavendishia bracteata* (Cabr) and *Clusia multiflora* (Clmu), were positioned on the right side of the first axis. Nevertheless, litter species occupied different positions in the multivariate functional space, indicating a diversity of combinations between anatomical traits.

### Leaf anatomy and decomposability at the community level

We estimated decay rates for each of the 63 species through a multiple regression analysis based on SLA (cm^2^ g^−1^), LNC (mg g^−1^), LCC (mg g^−1^), and A_max_ (nmol g^−1^ s^−1^). These four traits predicted 71% of the variation in decay rates of the 15 species from the decomposition experiment (P = 0.009, r = 0.84, r^2^ = 0.71, n = 15, Table S3).  Following, we calculated community-level decomposability per plot to estimate the relationships between CWM K rate y^−1^ and CWM of each leaf anatomical trait. This modelled CWM K rate y^−1^ showed and average of 0.42 ± 0.13_,_ ranging from 0.22 to 0.70.

**Fig. 3:**
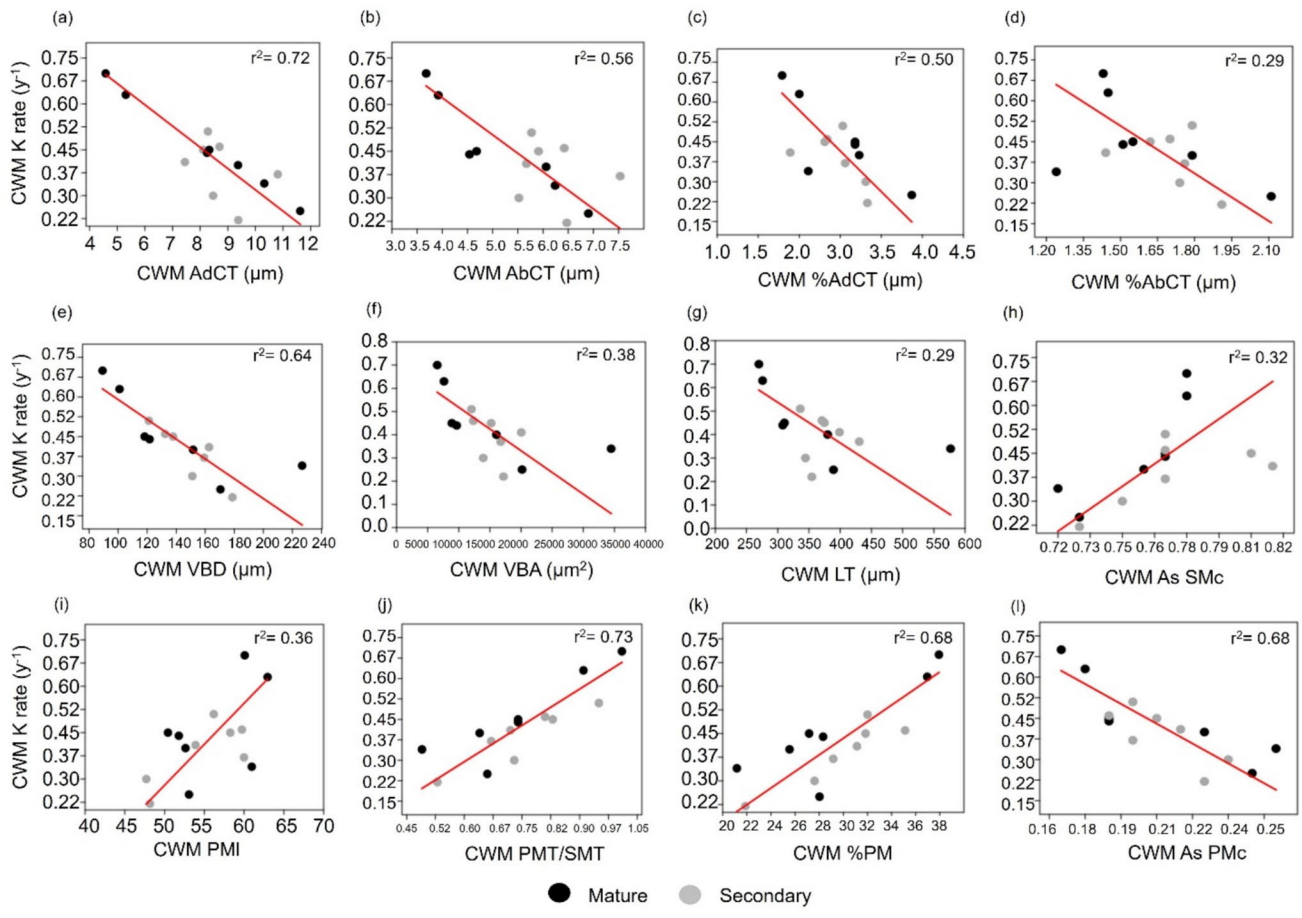
Reduced major axis regressions between Community Weighed Mean of decomposability (CWM K rate y^−1^) and CWM of **a** Adaxial cuticle thickness (CWM AdCT, µm), **b** Abaxial cuticle thickness (CWM AbCT, µm), **c** %Adaxial cuticle thickness (CWM %AdCT), **d** %Abaxial cuticle thickness (CWM %AbCT), **e** Vascular bundle diameter (CWM VBD, µm), (f) Vascular bundle area (CWM VBA, µm^2^), **g** Leaf thickness (CWM LT, µm), **h** Aspect ratio Spongy mesophyll cells (CWM As SMc), **i** Pallisade mesophyll cell length (CWM PMl, µm), **j** Pallisade mesophyll/ Spongy mesophyll ratio (CWM PMT/SMT), **k** %Pallisade mesophyll (CWM %PM), and **l** Aspect ratio Pallisade mesophyll cells (CWM As PMc). Dark dots represent mature forests and grey dots depict secondary forests. In the upper right corner of each plot the size effect is presented (r^2^). Red lines indicate significant relations between variables (P < 0.05)

Similar to the species Spearman correlation analysis, we found strong negative relationships between modelled CWM K rate y^−1^ and traits measured for all 63 species for: CWM AdCT (P = 0.00011, r^2^ = 0.72, n = 14, CI = [-0.08, -0.04]), CWM AbCT (P = 0.0021, r^2^ = 0.56, n = 14, CI = [− 0.16, − 0.08]), CWM %AdCT (P = 0.0046, r^2^ = 0.50, n = 14, C I = [− 0.42, 0.19]), CWM %AbCT (P = 0.047, r^2^ = 0.29, n = 14, CI = [− 1.51, − 0.26]), CWM VBD (P = 0.00061, r^2^ = 0.64, n = 14, CI = [− 0.005, − 0.002]), CWM VBA (P = 0.018, r^2^ = 0.38, n = 14, CI = [− 0.00002, − 0.0000007]), CWM LT (P = 0.046, r^2^ = 0.29, n = 14, CI = [− 0.003, 0.0002]), and CWM As PMc (P = 0.00031, r^2^ = 0.68, n = 14, CI = [− 6.53, − 2.97]). In contrast, significant positive relationships were observed between CWM K rate y^−1^ and CWM As SMc (P = 0.036, r^2^ = 0.32, n = 14, CI = [1.30, 7.06]), CWM PMl (P = 0.022, r^2^ = 0.36, n = 14, CI = [0.02, 0.04]), CWM PMT/SMT (P = 0.00010, r^2^ = 0.73, n = 14, CI = [0.58, 1.18]), and CWM %PM (P = 0.00031, r^2^ = 0.68, n = 14, CI = [0.02, 0.04]) (Fig. 3).

### Functional diversity and decomposability along succession

**Fig. 4:**
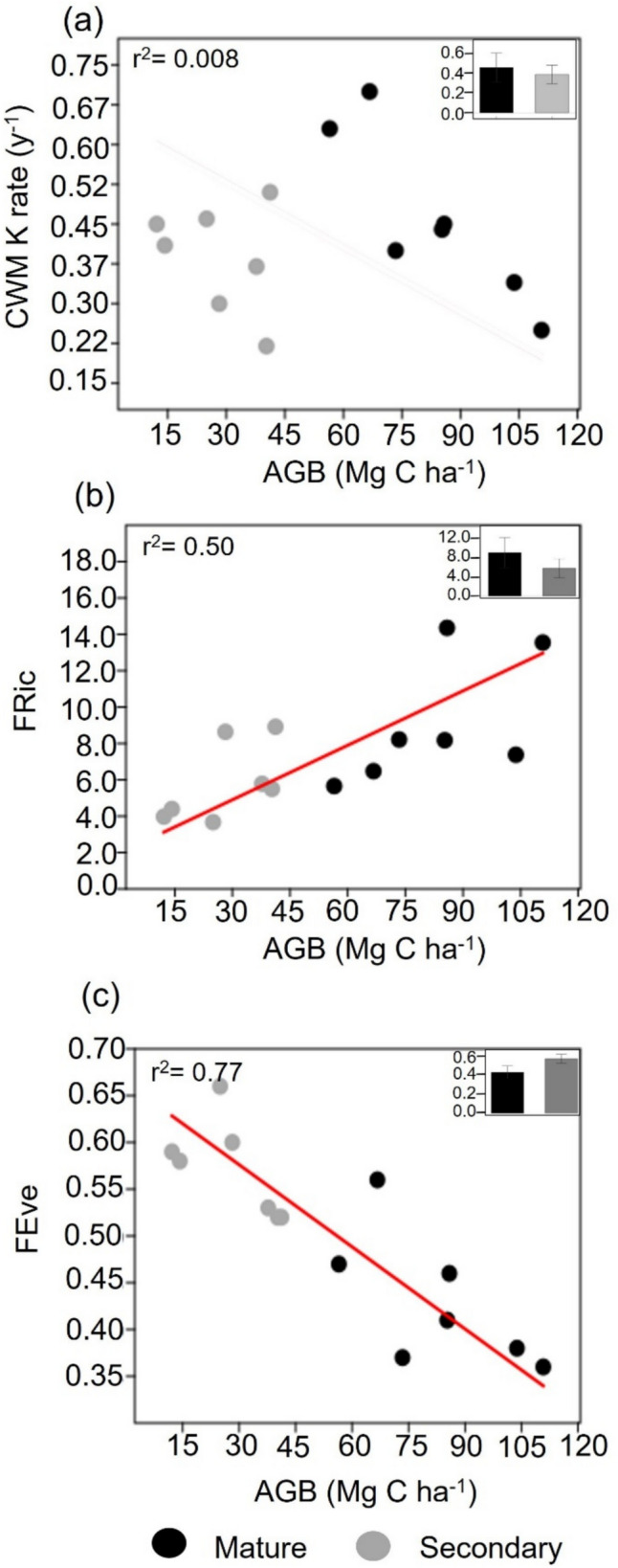
Reduced major axis regressions between aboveground biomass (Mg C ha^−1^) as a proxy of succession and **a** Community Weighed Mean of decomposability (CWM K rate y^−1^) **b** functional richness (FRic) and **c** functional evenness (FEve). In the upper left corner of each plot the size effect is presented (r^2^). In the upper right corner,

Based on the multiple regression model with the anatomical traits that best predicted decay rates at the community level (i.e., CWM PMT/SMT and CWM %AbCT, r^2^ = 0.89, Table 2), we calculated functional richness, functional evenness, functional divergence, and functional dispersion. Although CWM K rate y^−1^ (P = 0.761, r^2^ = 0.008, n = 14, CI = [− 0.02, − 0.003]), functional divergence (P = 0.313, r^2^ = 0.08, n = 14, CI = [− 0.02, − 0.002]), and functional dispersion (P = 0.905, r^2^ = 0.001, n = 14, CI = [− 0.03, 0.005]) did not correlate with AGB, we found that functional richness significantly increased with increasing AGB (P = 0.0046, r^2^ = 0.50, n = 14, CI = [0.06, 0.14]), while functional evenness significantly decreased with AGB (P < 0.0001, r^2^ = 0.77, n = 14, CI = [− 0.004, − 0.002]) (Fig. 4).

**Table 2:** Results from stepwise multiple linear regression model applied to decay rates (CWM K rate y^−1^). The following covariates were considered but not included: CWM AdCT, CWM %PMT, CWM %AdCT, CWM As SMc, CWM As PMc, CWM PMl, CWM AbCT, CWM VBA, CWM VBD, and CWM LT. VIF represents the Variance Inflation Factor

Model		Unstandardized	Standard Error	Standardized	t	P	Collinearity Statistics	
							Tolerance	VIF
Community level								
1	(Intercept)	0.424	0.035		11.996	< .001		
2	(Intercept)	– 0.142	0.101		– 1.405	0.185		
	CWM.PMT/SMT	0.760	0.134	0.854	5.689	< .001	1.000	1.000
3	(Intercept)	0.295	0.124		2.370	0.037		
	CWM.PMT/SMT	0.701	0.088	0.788	7.946	< .001	0.974	1.026
	CWM %AbCT	– 0.239	0.058	– 0.411	– 4.147	0.002	0.974	1.026

Model summary: r = 0.95, r^2^ = 0.89, Adjusted r^2^ = 0.88, RMSE = 0.047, P = 0.002

## Discussion

Our study consistently demonstrates that multiple leaf anatomical traits are strong predictors of litter decomposability at both species and community levels in tropical Andean montane forests. In addition to the well-known ‘soft’ leaf functional traits, we show that ‘hard’ anatomical traits including cuticles, mesophyll tissues, and vascular bundles, are highly correlated with decay rates. However, community-level decomposability showed no significant variation along the successional gradient, likely due to the counterbalancing effects of different dimensions of functional diversity, suggesting that distinct dynamics in community structure influence decay rates in secondary and mature forests.

### Leaf anatomy and its connection to species decomposability

Our first hypothesis (H1) was supported as we found strong correlations between litter decomposability of individual species and fractions of tissue types from mesophyll, vascular bundles, and cuticle, with the exception of the epidermis. Specifically, we found significant negative correlations between decay rates and both abaxial and adaxial cuticle thickness (Fig. 1a), which could be attributed to the mechanical and chemical resistance that the cuticle provides to the leaf. Acting as the outer protective layer of leaves, cuticles are composed of recalcitrant compounds like cutins, waxes, and polysaccharides, playing a fundamental role in shielding leaves from damage caused by different environmental and biotic stressors, such as wind, insects, pathogens, and microbial decomposers (Müller 2006; Riederer and Mülller 2006; Onoda et al. 2012). Thicker cuticles offer an important advantage in enhancing resistance due to their greater structural thickness and the strength of their constituent materials, resulting in longer leaf lifespans and a deceleration of decay rates (Onoda et al. 2012). Indeed, leaves with high cuticle thickness in both abaxial and adaxial side are strongly related to LMA (AdCT: P = 0.00019, r^2^ = 0.67, n = 15, CI = [2.20, 14.97], AbCT: P = 0.0012, r^2^ = 0.57, n = 15, CI = [6.40, 24.01]), which defines the plant ecological strategy along the LES (Fig. 2, Wright et al. 2004). Furthermore, cutins decompose at a slower rate compared to other proteins and carbohydrates, and they exhibit long retention times in soil (Gallardo and Merino 1993; Goni and Hedges 1995; Onoda et al. 2012). This is mainly attributed to cutins being more resistant to chemical degradation (Schreiber and Schönherr 2009), and given that the cuticle constitutes a significant fraction of leaf dry mass in plant species (Onoda et al. 2012), this anatomical structure appears to be crucial in determining the role of decomposition in ecosystem carbon cycling. In this sense, our findings corroborate the insights of Onoda et al. (2012), who emphasized the potential significant role of cuticle thickness in shaping carbon turnover and accumulation in ecosystems.

Contrary to our initial expectations, we found no correlation between the epidermis, expressed as thickness or a proportion of total leaf size, with decay rates in either the adaxial or abaxial layer (Fig. 1a). While the leaf epidermis is traditionally recognized for its role in providing a protective barrier against mechanical injury, pathogens, and UV radiation, this tissue serves to other functions, including water conservation, osmoregulation, secretion of substances, reflecting and absorbing light, structural support, and even sensory functions (Dietz and Hartung 1996; Glover 2000; Javelle et al. 2011; Cavé-Radet et al. 2020; Galviz and Valerio 2021). Given that the epidermis is the outermost layer of cells interacting with a plenty of environmental variables, and has multiple functions, its variations may respond to different trade-offs, potentially weakening its relationship with decay rates.

Significant negative associations were also observed between vascular bundle size and decay rates (Fig. 1a). This finding can be explained due to the direct deposition of lignin within the cell walls of xylem and phloem tissues (Barros et al. 2015; Maceda et al. 2021), which facilitates long-distance water transport through the plant (Ménard et al. 2022). Consequently, plants with larger diameters and increased vascular bundle areas contain a substantial proportion of this recalcitrant polyphenolic polymer, making it difficult to degrade. Collectively, the presence of larger vascular bundles and thicker cuticles suggests that traits related to water use efficiency and mechanical resistance are integral components of plant ecological strategies (Fig. 2), despite the higher construction costs associated with them (Xing et al. 2021; Ni et al. 2022). These strategies lead to extended leaf lifespans and slower decay rates.

Even though absolute palisade thickness showed no significant correlation with decay rates, the proportion of palisade tissue (%) and its ratio to spongy tissue thickness exhibited a strong positive relationship with decay rates (Fig. 1). This result aligns with our initial hypothesis (H1), as palisade tissues contain chloroplasts with high concentration of nitrogen allocated to the photosynthetic machinery (Evans 1989; Terashima et al. 2011; Onoda et al. 2017). Consequently, leaves with a high percentage of palisade tissue likely increase the litter quality, thus becoming particularly attractive to decomposers. This effect is likely to be magnified in ecosystems with pronounced nitrogen constraints, such as the tropical Andean montane forests (Vitousek et al. 1988; Tanner et al. 1998; Wilcke et al. 2008; Myster 2021), where decomposers actively seek out leaves with elevated nitrogen content due to its essential role in their metabolism (Bakker et al. 2011) and its scarcity in such environments (Vitousek et al. 1988; Tanner et al. 1998; Soethe et al. 2008). Interestingly, we also observed a negative relationship between the width of palisade cells and decay rates (Fig. 1), suggesting that the shape of palisade cells may be linked to decomposability. The development of elongated and cylindrical cells is promoted by phototropins triggered by blue light (Watson 1942; Kozuka et al. 2011). This unique cell shape maximizes photosynthesis by aligning perpendicularly to the epidermis (Terashima and Saeki 1983; Terashima et al. 2006), which could potentially allocate more resources for decomposers. In line with this idea, we found a positive relation between palisade long and its thickness (P = 0.01, rho = 0.63, n = 15). This effect is also consistent at the community level (see below).

### Community-level relationships between leaf anatomy and decomposability

We confirm the same results observed at the species level concerning the negative correlations with cuticles and vascular bundles and the positive ones with palisade mesophylls, supporting our second hypothesis (H2, Fig. 1 and 4). However, at the community level, we also found significant relationships with other anatomical structures. In particular, higher leaf anatomical thickness is negatively associated with decay rates (Fig. 3g). Thicker leaves are also related to a higher LMA at both species (P = 0.002, rho = 0.72, n = 15) and community levels (P = 0.002, rho = 0.75, n = 14), which, in turn, can decrease decay rates (Wright et al. 2004; Onoda et al. 2012; Wang et al. 2025). Higher leaf thickness may result from increased mass investment in leaf tissues, which consequently increases LMA (John et al. 2017), as our findings show that leaf thickness is positively correlated with the thickness of nearly all tissues (Fig. 1). Considering that studies in the upper Andean tropical forests have indicated that plant communities tend to exhibit more conservative strategies, characterized by leaves with high leaf thickness, high leaf toughness, and high LDMC (Homeier et al. 2021; Báez et al. 2022; Martínez-Villa et al. 2024), it is likely that these traits are also related to thicker leaf anatomical structures.

Interestingly, we found a positive relationship between the aspect ratio of spongy cells (CWM As SMc) and modelled community decay rates (CWM K rate y^−1^), which means that circular spongy cells are related to faster litter breakdown. The spongy mesophyll is related to gas diffusion within the leaf and a thicker spongy mesophyll with larger intercellular spaces is assumed to enhance gas exchange (Terashima et al. 2011; He et al. 2018; Ni et al. 2022). The spongy mesophyll is also a photosynthetic tissue and cells with circular shapes likely maximize the surface area for gas exchange with the leaf intercellular spaces. Thus, rounder cells, which can allocate higher nitrogen concentrations, may be associated with a more acquisitive strategy that prioritizes maximizing photosynthesis, which is, in turn, positively correlated with decay rates (Fig. 3).

Given that spongy and palisade tissues were inversely correlated (Fig. 1b) and higher palisade/mesophyll ratio increases decay rates at both species (Fig. 1c) and community levels (Fig. 3j, Table 2), the mechanisms underlying the ratio between these mesophylls are needed to be further studied. One potential explanation is that a higher investment in spongy mesophyll can optimize photosynthesis by compensating for a lower investment in palisade tissue, thus effectively capturing scattered light, and this balance can vary depending on environmental conditions. In line with this idea, a recent study by Ni et al. (2022) reveals a trade-off in plants: species with thin leaves and a high palisade mesophyll optimize photosynthesis in higher latitudes and short growing seasons, while those with thick leaves and a high spongy mesophyll are better suited in lower latitudes under shaded and more scattered light conditions. Collectively, all these findings suggest that the dynamic balance between spongy and palisade mesophylls may shape crucial ecological functions, but further empirical evidence is needed to support this hypothesis.

### Changes of functional diversity and decomposability along the successional gradient

When examining functional diversity along our gradient of forest succession, we observed that old-growth forests encompassed a wider functional richness but lower evenness for traits significantly related to decomposability, and that decay rates did not change along succession contrary to our third hypothesis (H3, Fig. 4). Higher functional richness indicates that the range of values of anatomical traits related to decomposability increase as succession progresses, suggesting the occupation of various niches with the arrival of different species. The increase of species richness and the change in species composition with succession have long been demonstrated in tropical forests (Chazdon et al. 2014; Rozendaal et al. 2019; Poorter et al. 2021a; Elsy et al. 2024). This is particularly evident for rare species with new traits, which become more prevalent as succession advances (Elsy et al. 2024), thus contributing to increased functional richness (van der Sande et al. 2024). Nonetheless, in the later stages of succession, low functional evenness suggests that dominant plant species are distributed within specific combinations of leaf anatomical traits, unlike in the earlier stages. In accordance with our results, a comprehensive continental study of successional patterns in functional diversity has highlighted that functional evenness tends to decrease with succession primarily due to biotic processes such as competition (van der Sande et al. 2024). This results in the convergence of similar trait values of dominant species, while simultaneously allowing for the coexistence of a wide variety of rare species with distinctive functional traits (van der Sande et al. 2024). In our study, the opposite trends between functional evenness and functional richness could cancel out any discernible pattern of decay rates along the successional gradient (Fig. 4). Higher functional evenness in early stages of succession may increase complementarity effects on decomposability within a constrained functional space. This is consistent with other studies that have shown complementarity effects on litter decomposition (García-Palacios et al. 2017) and nutrient cycling (Kahmen et al. 2006). By contrast, in mature forests, AGB is distributed in few dominant plant species with conservative traits, thereby increasing the functional redundancy, despite exhibiting a broader range of anatomical traits linked to decomposability in the whole community.

### Limitations of the study

Although few studies have linked anatomical traits to decomposition, our findings highlight their significant role in this process by showing their association with species-level decomposability, supporting their inclusion in trait-based decomposition frameworks. However, a key limitation of our study is the potential for circular reasoning, given that foliar and anatomical traits—although distinct—are often correlated within the leaf economic spectrum (Somavilla et al. 2014; Harrison et al. 2021). While we used species-level foliar traits to estimate the decomposition rates of remaining species in the community, we also examined how anatomical traits relate to community-level decomposability. This raises concerns about potential circularity, although we assessed independently measured traits across ecological scales rather than testing the same variable against itself. Ideally, direct decomposition measurements for all species in the community—though requiring substantial experimental effort—would be preferable to estimations. While our approach provides a coherent and widely accepted estimation of decomposition rates according to the literature (Santiago 2007; Bakker et al. 2011; de la Riva et al. 2019), its reliance on indirect measures based on foliar traits may introduce some imprecision. Future studies should address these limitations by incorporating direct decomposition measurements for all the species, expanding trait datasets, and employing more extensive experimental approaches to validate these associations.

Another important consideration is the use of CWM and functional diversity metrics, which are influenced by community structure. While this could introduce interdependence in our regression models, these metrics capture different ecological dimensions: CWM reflects the dominant functional strategy of the community (Garnier et al. 2004, 2016), whereas functional diversity indices describe the range and distribution of traits (Villéger et al. 2008; Schmera et al. 2023), providing insights into complementarity and redundancy. Despite these potential constraints, our approach allowed us to explore how shifts in community trait composition might shape decomposition patterns along succession.

Finally, while we demonstrated that leaf anatomy is a central factor influencing decay rates at both species and community levels, litter decomposition is a complex process shaped by multiple factors, including abiotic conditions (Salinas et al. 2011), litter quality (Castillo-Figueroa 2024a; Castillo-Figueroa et al. 2025), decomposer communities (Castillo-Figueroa and Castillo-Avila 2025), and non-additive effects (Castillo-Figueroa 2024b), all of which can change along succession. Although these aspects are beyond the scope of this study, examining their interplay could further enhance our understanding of decomposition processes along successional gradients.

## Conclusions

Our study integrates leaf anatomy into litter decomposability, revealing that traits such as cuticles, vascular bundles, palisade and spongy mesophylls play a pivotal role in predicting litter breakdown and may substantially influence carbon turnover in the ecosystems. Additionally, we found a strong correlation between leaf anatomical traits and decay rates at the community level. Specifically, plant communities with thicker protective leaf structures such as cuticles and larger vascular tissues displayed lower decay rates. Conversely, a higher proportion of palisade tissues relative to spongy tissues, along with a higher prevalence of cylindrical cells in palisade mesophylls and circular cells in spongy mesophylls, are related to increase decay rates. Remarkably, as succession progresses, predicted decay rates did not exhibit significant shifts. This finding could be explained by a balance between higher functional evenness in secondary forests within a narrow functional space, and lower functional evenness in mature forests within a broader functional space in the attributes linked to decomposability. This suggests that in tropical Andean montane forests, which are recognized as global carbon sinks, the dynamics driving functional diversity of leaf anatomical traits related to decomposability within plant communities vary between secondary and mature forests, thereby affecting the influence of succession on decay rates. Further studies should address limitations in circularity, interdependence between functional diversity metrics, and other biotic and abiotic factors varying along succession.

## Supplementary Information

Below is the link to the electronic supplementary material.

Supplementary file1 (DOCX 683 kb)

## Data Availability

The datasets generated and/or analyzed during the current study are openly available in the Open Science Framework repository at: https://osf.io/afxmv/.
