# Supplementary material for: Are leaf anatomical traits strong predictors of litter decomposability? Evidence from upper Andean tropical species along a forest successional gradient
Source: Oecologia. 2025 Jun 24;207(7):110. doi: 10.1007/s00442-025-05739-8 (PMC12187801; doi:10.1007/s00442-025-05739-8)
Supplement: Supplementary file 1 — Supplementary file1 (DOCX 683 kb) [file 442_2025_5739_MOESM1_ESM.docx]

Supplementary information

**Are leaf anatomical traits strong predictors of litter decomposability? Evidence from upper Andean tropical species along a forest successional gradient**

Dennis Castillo-Figueroa^1*^, Juan M. Posada^1^

^1^Biology Department, Faculty of Natural Sciences, Universidad Del Rosario, Bogotá, Colombia.

*Corresponding author, Email: [dennis.castillof@gmail.com](mailto:dennis.castillof@gmail.com) Number: 571 3195881103

ORCID:

DC-F: <https://orcid.org/0000-0002-4584-0762>

JP: <https://orcid.org/0000-0001-7794-9300>

**Figure S1.** Study area where this study was conducted. The grey triangles indicate each of the four study sites in upper Andean tropical forests.

**
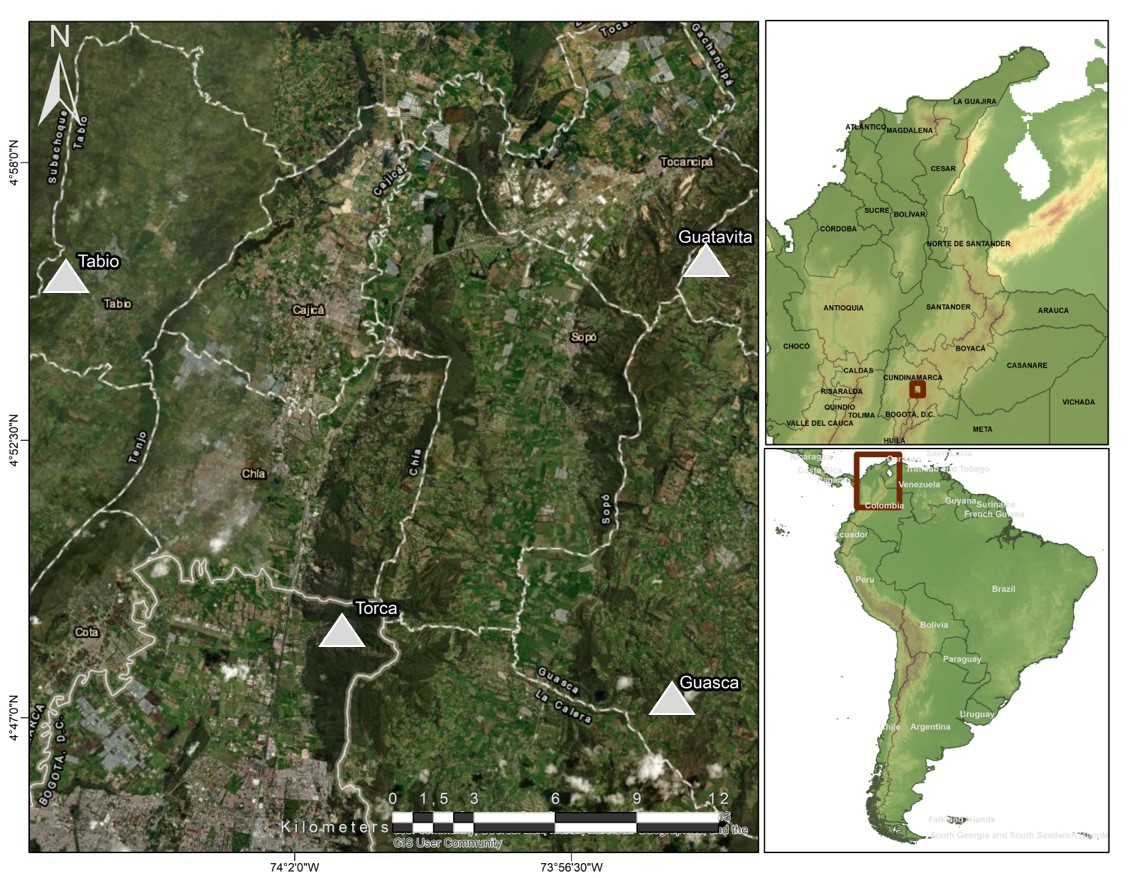
**

**Figure S2.** Light microscopy image of leaf transverse profile in *Clusia multiflora* showing the different anatomical traits measured in this study. Measurements on tissue sections (a) comprise the following acronyms: AdCT: Adaxial cuticle thickness (µm), AbCT: Abaxial cuticle thickness (µm), AdET: Adaxial epidermis thickness (µm), AbET: Abaxial epidermis thickness (µm), AdHT: Adaxial hipodermis thickness (µm), PMT: Pallisade mesophyll thickness (µm), SMT: Spongy mesophyll thickness (µm), AS: Air space (µm^2^), VBA: Vascular bundle area (µm^2^), VBD: Vascular bundle diameter (µm), LT: Leaf thickness (µm). Measurements on cell types (b) comprise the following acronyms: AdEw: Adaxial epidermis cell width (µm), AdEl: Adaxial epidermis cell long (µm), AdHw: Adaxial hypodermis cell width (µm), AdHl: Adaxial hypodermis cell long (µm), PMw: Pallisade mesophyll cell width (µm), PMl: Pallisade mesophyll cell long (µm), SMw: Spongy mesophyll cell width (µm), SMl: Spongy mesophyll cell long (µm), AbEw: Abaxial epidermis cell width (µm), and AbEl: Abaxial epidermis cell long (µm).


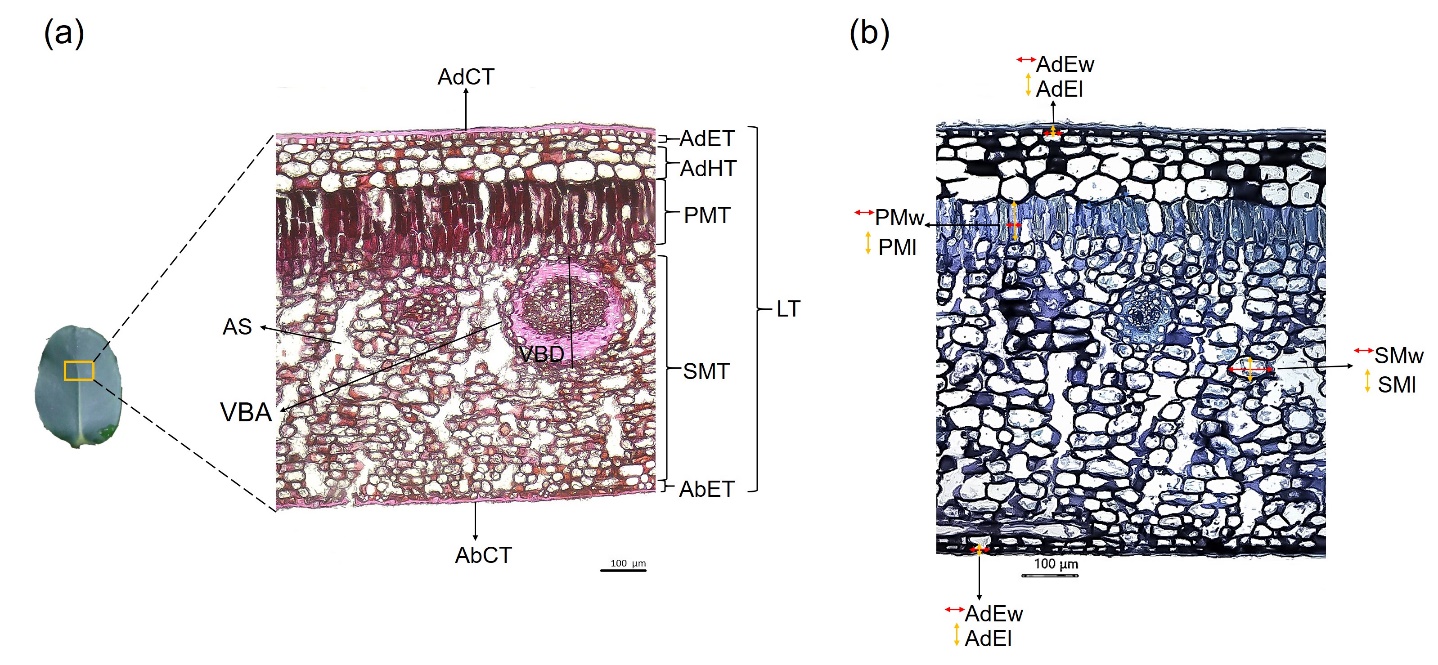


**Table S1.** Species dominance in the 14 permanent plots were this study was conducted.

| **Plot** | **Number of species** | **Number of total individuals** | **Species with highest dominance (ind, %)** |
| --- | --- | --- | --- |
| 1 | 19 | 172 | *Myrcianthes leucoxyla* (63, 36.63%) |
|  |  |  | *Miconia squamulosa* (24, 13.95%) |
|  |  |  | *Macleania rupestris* (20, 11.63%) |
|  |  |  | *Vallea stipularis* (12, 6.98%) |
|  |  |  | *Miconia ligustrina* (11, 6.40%) |
| 2 | 14 | 144 | *Miconia squamulosa* (32, 22.22%) |
|  |  |  | *Myrcianthes leucoxyla* (26, 18.06%) |
|  |  |  | *Macleania rupestris* (16, 11.11%) |
|  |  |  | *Miconia ligustrina* (15, 10.41%) |
|  |  |  | *Vallea stipularis* (15, 10.42%) |
| 3 | 16 | 119 | *Weinmania tomentosa* (74, 62.18%) |
|  |  |  | *Cavendishia bracteata* (7, 5.88%) |
|  |  |  | *Myrsine coriácea* (6, 5.04%) |
|  |  |  | *Miconia ligustrina* (6, 5.04%) |
|  |  |  | *Bejaria resinosa* (6, 5.04%) |
| 4 | 11 | 179 | *Cavendishia bracteata* (120, 67.04%) |
|  |  |  | *Gaiadendron punctatum* (24, 13.41%) |
|  |  |  | *Alnus acuminata* (10, 5.59%) |
|  |  |  | *Weinmania tomentosa* (8, 4.47%) |
|  |  |  | *Miconia ligustrina* (5, 2.80%) |
| 5 | 22 | 92 | *Weinmania tomentosa* (37, 40.22%) |
|  |  |  | *Viburnum triphyllum* (10, 10.87%) |
|  |  |  | *Myrsine guianensis* (8, 8.79%) |
|  |  |  | *Myrsine coriácea* (7, 7.61%) |
|  |  |  | *Cavendishia bracteata* (5, 5.44%) |
| 6 | 15 | 186 | *Cavendishia bracteata* (70, 37.63%) |
|  |  |  | *Gaiadendron punctatum* (26, 13.98%) |
|  |  |  | *Alnus acuminata* (25, 13.44%) |
|  |  |  | *Myrsine coriacea* (13, 6.99%) |
|  |  |  | *Diplostephium rosmarinifolum* (10, 5.38%) |
| 7 | 21 | 219 | *Miconia squamulosa* (54, 24.66%) |
|  |  |  | *Daphnopsis caracasana* (31, 14.16%) |
|  |  |  | *Myrcianthes leucoxyla* (29, 13.24%) |
|  |  |  | *Ilex kunthiana* (19, 8.68%) |
|  |  |  | *Cavendishia bracteata* (14, 6.39%) |
| 8 | 13 | 196 | *Myrsine guianensis* (60, 30.61%) |
|  |  |  | *Daphnopsis caracasana* (34, 17.35%) |
|  |  |  | *Miconia squamulosa* (23, 11.73%) |
|  |  |  | *Morela parifolia* (20, 10,.21%) |
|  |  |  | *Viburnum triphyllum* (12, 6.12%) |
| 9 | 19 | 76 | *Miconia squamulosa* (22, 28.95%) |
|  |  |  | *Croton bogotanus* (10, 13.16%) |
|  |  |  | *Xylosma spiculifera* (9, 11.84%) |
|  |  |  | *Piper bogotensis* (5, 6.58%) |
|  |  |  | *Daphnopsis caracasana* (4, 5.26%) |
| 10 | 18 | 148 | *Viburnum triphyllum* (39, 26.35%) |
|  |  |  | *Miconia squamulosa* (22, 14.86%) |
|  |  |  | *Daphnopsis caracasana* (13, 8.78%) |
|  |  |  | *Critoniopsis bogotanus* (12, 8.11%) |
|  |  |  | *Duranta mutissi* (11, 7.43%) |
| 11 | 16 | 70 | *Clusia multiflora* (14, 20.00%) |
|  |  |  | *Drimys granadiensis* (14, 20.00%) |
|  |  |  | *Weinmania tomentosa* (7, 10.00%) |
|  |  |  | *Cavendishia nitida* (7, 10.00%) |
|  |  |  | *Ilex kunthiana* (5, 7.15%) |
| 12 | 15 | 148 | *Weinmania tomentosa* (64, 43.24%) |
|  |  |  | *Clusia multiflora* (17, 11.49%) |
|  |  |  | *Bejaria resinosa* (16, 10.81%) |
|  |  |  | *Cavendishia nitida* (12, 8.11%) |
|  |  |  | *Myrsine coriacea* (9, 6.08%) |
| 13 | 22 | 133 | *Miconia squamulosa* (43, 32.33%) |
|  |  |  | *Viburnum triphyllum* (21, 15.79%) |
|  |  |  | *Palicourea lineariflora* (13, 9.77%) |
|  |  |  | *Palicourea angustifolia* (11, 8.27%) |
|  |  |  | *Oreopanax incisus* (6, 4.51%) |
| 14 | 15 | 158 | *Clusia multiflora* (51, 32.28%) |
|  |  |  | *Weinmania tomentosa* (46, 29.11%) |
|  |  |  | *Bejaria resinosa* (13, 8.23%) |
|  |  |  | *Ilex kunthiana* (11, 6.96%) |
|  |  |  | *Cavendishia bracteata* (11, 6.96%) |
|  |  |  | *Cavendishia nitida* (9, 5.70%) |

**Table S2.** List of the 63 plant species in the 14 permanent plots and their scores to the first axis of the PCA based on litter functional traits. PCA was conducted based on seen traits: SLA= Specific Leaf Area, cm^2^/g, LA= Leaf Area, cm^2^, LDMC= Leaf Dry Matter Content, mg/g, LCC= Leaf Carbon Content, mg/g, LNC= Leaf Nitrogen content, mg/g, LT= Litter thickness, mm, LD= Leaf Density, mg/mm^3^. Positive scores are related to conservative strategy, while negative scores are associated to acquisitive strategy. Biplot can be found in Castillo-Figueroa et al. (2025). In **red** the 15 species selected for the litter decomposition experiment.

| **Species names** | **PC 1 (Functional spectrum)** |
| --- | --- |
| *Abatia parviflora* | -0.73718 |
| *Ageratina asclepiadea* | 0.085956 |
| *Ageratina fastigiata* | 0.099112 |
| *Ageratina glyptophlebia* | -0.98637 |
| ***Alnus acuminata*** | **-0.71739** |
| *Aiouea dubia* | 1.012 |
| *Baccharis macrantha* | -0.42052 |
| *Barnadesia spinosa* | -1.468 |
| *Bejaria resinosa* | 0.43554 |
| *Bucquetia glutinosa* | -0.21947 |
| ***Cavendishia bracteata*** | **1.00820** |
| *Cavendishia nitida* | 1.37300 |
| ***Cedrela montana*** | **-0.17349** |
| *Citharexylum sulcatum* | 0.65399 |
| *Clethra fimbriata* | 0.75867 |
| *Clethra lanata* | 0.39773 |
| ***Clusia multiflora*** | **0.49431** |
| *Critoniopsis bogotana* | -0.57534 |
| ***Croton bogotanus*** | **-1.4909** |
| *Cybianthus iteoides* | -0.18529 |
| ***Daphnopsis caracasana*** | **-0.2566** |
| *Diplostephium rosmarinifolium* | 0.013333 |
| ***Drymis granadiensis*** | **0.31206** |
| *Duranta mutisii* | 1.1189 |
| *Escallonia discolor* | -0.45763 |
| *Gaiadendron punctatum* | 0.80424 |
| *Hedyosmum sp* | -0.61753 |
| *Hesperomeles goudotiana* | 0.99902 |
| ***Ilex kunthiana*** | **1.2999** |
| *Lippia hirsuta* | -1.4769 |
| *Macleania rupestris* | 0.66634 |
| *Macrocarpaea glabra* | -1.6557 |
| *Maytenus laxiflora* | 0.86623 |
| *Miconia elaeoides* | -0.57614 |
| *Miconia ligustrina* | -0.16268 |
| ***Miconia squamulosa*** | **0.082214** |
| ***Morella parvifolia*** | **0.62294** |
| *Morella pubescens* | 0.26399 |
| *Myrcianthes leucoxyla* | 1.6843 |
| ***Myrsine coriacea*** | **0.89023** |
| *Myrsine dependens* | -0.17641 |
| *Myrsine guianensis* | 1.0157 |
| ***Ocotea calophylla*** | **0.74866** |
| *Oreopanax bogotensis* | 0.91918 |
| *Oreopanax incisus* | -0.0032272 |
| *Palicourea angustifolia* | -0.32357 |
| *Palicourea demissa* | -0.45296 |
| *Palicourea lineariflora* | -0.3288 |
| ***Piper bogotense*** | **-1.7624** |
| ***Prunus buxifolia*** | **1.8857** |
| *Psychotria boqueronensis* | -0.5217 |
| *Rhamnus goudotiana* | -0.54881 |
| *Rhamnus sphaerosperma* | -0.42132 |
| *Sessea corymbosa* | 0.17135 |
| *Symplocos theiformis* | 1.4416 |
| *Ulex europaeus* | 0.067323 |
| ***Vallea stipularis*** | **-0.6534** |
| *Varronia cylindristachya* | -0.91304 |
| *Vasconcellea pubescens* | -3.7274 |
| *Verbesina arborea* | -2.0100 |
| *Viburnum triphyllum* | -0.12204 |
| *Weinmannia tomentosa* | 0.97359 |
| *Xylosma spiculifera* | 0.97719 |

Castillo-Figueroa D, Soler-Marín D, Posada J (2025) Functional traits and species identity drive decomposition along a successional gradient in upper Andean tropical forests. Biotropica 57: e13425 <https://doi.org/10.1111/btp.13425>

**Table S3.** Multiple regression analysis that predict decay rates (P = 0.009, r = 0.84, r^2^ = 0.71) in the 15 plant species from the decomposition experiment. This model includes four traits: A_max_, LNC, LCC and SLA. Decay rates from the 48 remaining species were estimated based on the coefficients and the intercept of the model.

|  | **Coefficients** | **Standard error** | **t** |
| --- | --- | --- | --- |
| Intercept | 1.14630768 | 1.305887 | 0.87780006 |
| A_max_ (μmol m^-2^ s^-1^) | -0.00016664 | 0.00944654 | -0.01764051 |
| LNC (mg g^-1^) | 0.01013454 | 0.02898577 | 0.34963838 |
| LCC (mg g^-1^) | -0.00244339 | 0.00200922 | -1.2160892 |
| SLA (cm^2^ g^-1^) | 0.00516302 | 0.00554969 | 0.93032652 |

**Table S4.** Descriptive statistics of the anatomical traits of the 15 plant species included in the litter decomposition experiment. The average and standard deviation of the remaining 48 species are also shown. Acronyms can be found in Table 1.

| **Species** | **AdCT (µm)** | **AbCT (µm)** | **AdET (µm)** | **AbET (µm)** | **AdHT (µm)** | **PMT (µm)** | **SMT (µm)** | **AS (µm^2^)** | **VBA (µm^2^)** | **VBD (µm)** | **LT (µm)** |
| --- | --- | --- | --- | --- | --- | --- | --- | --- | --- | --- | --- |
| *Alnus acuminata* | 4.21 ±0.84 | 2.78 ± 0.45 | 8.70 ± 1.45 | 10.92 ± 1.91 | 17.54 ± 5.65 | 98.51 ± 8.58 | 63.37 ± 8.24 | 1003.39 ± 571.25 | 2479.95 ± 988.28 | 66.40 ± 13.05 | 206.90 ± 12.84 |
| *Cavendishia bracteata* | 10.25 ± 2.64 | 7.44 ± 1.54 | 23.75 ± 5.61 | 13.77 ± 1.97 | 32.55 ± 7.47 | 53.88 ± 7.47 | 176.49 ± 28.03 | 3879.14 ± 1912.62 | 19537.68 ± 13947.34 | 200.88 ± 81.40 | 332.97 ± 38.88 |
| *Cedrela montana* | 4.59 ± 1.22 | 3.20 ± 0.57 | 10.12 ± 3.50 | 7.38 ± 1.40 | 0.00 | 99.21 ± 10.67 | 105.58 ± 18.72 | 4755.53 ± 1659.42 | 5938.36 ± 1951.74 | 89.45 ± 14.93 | 235.82 ± 19.37 |
| *Clusia multiflora* | 10.70 ± 1.51 | 6.89 ± 1.75 | 13.46 ± 2.36 | 15.18 ± 6.18 | 111.73 ± 12.95 | 115.49 ± 40.60 | 567.48 ± 72.26 | 8202.49 ± 4782.09 | 59821.36 ± 23937.55 | 331.40 ± 89.50 | 832.06 ± 106.02 |
| *Croton bogotanus* | 2.68 ± 0.46 | 2.30 ± 0.45 | 10.21 ± 1.58 | 9.09 ± 2.87 | 23.99 ± 10.73 | 72.95 ± 13.47 | 56.90 ± 11.72 | 1265.72 ± 816.22 | 1756.91 ± 947.17 | 49.68 ± 14.19 | 157.62 ± 17.35 |
| *Daphnopsis caracasana* | 4.74 ± 1.07 | 4.72 ± 1.02 | 38.19 ± 5.17 | 22.58 ± 3.44 | 0.00 | 122.73 ± 23.64 | 247.38 ± 36.48 | 7304.86 ± 2698.97 | 10784.26 ± 4701.47 | 124.78 ± 30.22 | 449.25 ± 62.40 |
| *Drymis granadiensis* | 10.10 ± 1.54 | 10.44 ± 1.86 | 16.99 ± 2.88 | 18.02 ± 5.06 | 18.01 ± 4.03 | 53.79 ± 21.31 | 96.38 ± 22.18 | 3445.60 ± 1143.51 | 7038.63 ± 2679.94 | 108.98 ± 26.25 | 226.73 ± 31. 55 |
| *Ilex kunthiana* | 22.52 ± 3.55 | 13.14 ± 2.25 | 43.77 ± 11.03 | 18.06 ± 3.10 | 40.84 ± 10.87 | 174.97 ± 41.85 | 209.47 ± 25.28 | 6380.07 ± 2293.71 | 22663.36 ± 11273.61 | 191.64 ± 56.68 | 529.73 ± 24.16 |
| *Miconia squamulosa* | 5.87± 1.79 | 5.68 ± 1.49 | 13.50 ± 1.71 | 16.71 ± 4.60 | 24.72 ± 7.09 | 96.32 ± 10.32 | 129.00 ± 41.07 | 1438.68 ± 812.56 | 2844.96 ± 999.01 | 64.69 ± 12.69 | 304.20 ± 48.64 |
| *Morella parvifolia* | 7.15 ± 1.88 | 4.20 ± 0.61 | 12.55 ± 2.20 | 9.78 ± 1.69 | 0.00 | 84.01 ± 15.78 | 97.77 ± 17.70 | 5753.50 ± 1728.09 | 8343.10 ± 4227.85 | 116.93 ± 40.30 | 217.51 ± 34.93 |
| *Myrsine coriacea* | 9.49 ± 2.87 | 5.08 ± 1.15 | 16.08 ± 3.21 | 13.66 ± 2.64 | 0.00 | 70.65 ± 16.54 | 146.70 ± 55.03 | 4298.13 ± 1707.59 | 13086.38 ± 9328.39 | 146.45 ± 69.39 | 262.03 ± 61.54 |
| *Ocotea calophylla* | 9.43 ± 1.63 | 7.11 ± 1.64 | 19.88 ± 3.24 | 13.55 ± 2.08 | 32.11 ± 7.25 | 144.04 ± 9.65 | 176.98 ± 23.36 | 4373.68 ± 2280.03 | 28759.53 ± 19811.95 | 223.61 ± 64.57 | 402.67 ± 12.69 |
| *Piper bogotense* | 3.54 ± 0.56 | 2.37 ± 0.43 | 10.94 ± 2.45 | 10.79 ± 2.86 | 39.30 ± 6.65 | 73.97 ± 9.93 | 66.42 ± 13.04 | 702.88 ± 373.70 | 1287.80 ± 886.30 | 41.99 ± 13.96 | 206.60 ± 10.06 |
| *Prunus buxifolia* | 6.99 ± 1.18 | 4.99 ± 0.91 | 44.11 ± 15.12 | 17.78 ± 2.50 | 0.00 | 92.57 ± 10.58 | 174.65 ± 33.54 | 6104.71 ± 1993.01 | 17480.52 ± 11238.48 | 161.31 ± 64.02 | 340.63 ± 55.09 |
| *Vallea stipularis* | 3.68 ± 0.50 | 2.50 ± 0.37 | 16.85 ± 4.89 | 14.19 ± 2.72 | 0.00 | 105.76 ± 17.08 | 84.36 ± 20.43 | 1431.33 ± 1145.02 | 4103.69 ± 1831.47 | 81.87 ± 20.54 | 232.93 ± 33.60 |
| **15 Species** | 7.73 ± 5.08 | 5.52 ± 3.25 | 19.94 ± 13.05 | 14.10 ± 5.10 | 22.72 ± 28.89 | 97.26 ± 37.42 | 159.93 ± 126.4 | 4022.65± 3086.9 | 13728.43 ± 17840.92 | 133.34 ± 89.80 | 329.18 ± 173.09 |
| **48 remaining species** | 6.91 ± 4.27 | 4.65 ± 2.71 | 20.84 ± 8.34 | 12.37 ± 4.74 | 16.19 ± 27.54 | 95.37 ± 42.40 | 247.35 ± 105.70 | 3459.85 ± 3620.37 | 10909.36 ± 12188.22 | 121.68 ± 68.50 | 309.10 ± 159.53 |
